# Sulfated liposome-based artificial cell membrane glycocalyx nanodecoys for coronavirus inactivation by membrane fusion

**DOI:** 10.1016/j.bioactmat.2023.10.021

**Published:** 2023-11-04

**Authors:** Xu Li, Ningtao Cheng, Danrong Shi, Yutong Li, Chen Li, Miaojin Zhu, Qiao Jin, Zhigang Wu, Linwei Zhu, Yi He, Hangping Yao, Jian Ji

**Affiliations:** a MOE Key Laboratory of Macromolecule Synthesis and Functionalization, Department of Polymer Science and Engineering, Zhejiang University, Hangzhou, 310027, China; b School of Medicine, Zhejiang University, Hangzhou, 310058, China; c State Key Laboratory for Diagnosis and Treatment of Infectious Diseases, National Clinical Research Center for Infectious Diseases, Collaborative Innovation Center for Diagnosis and Treatment of Infectious Diseases, The First Affiliated Hospital, School of Medicine, Zhejiang University, Hangzhou, 310003, China; d College of Chemical and Biological Engineering, Zhejiang University, Hangzhou, 310027, China; e Shanxi-Zheda Institute of Advanced Materials and Chemical Engineering, Taiyuan, 030032, China

**Keywords:** Antiviral nanoparticle, Cell membrane nanodecoy, Coronavirus, Viral inactivation

## Abstract

As a broad-spectrum antiviral nanoparticle, the cell membrane nanodecoy is a promising strategy for preventing viral infections. However, most of the cell membrane nanodecoys can only catch virus and cannot induce inactivation, which may bring about a considerably high risk of re-infection owing to the possible viral escape from the nanodecoys. To tackle this challenge, sulfated liposomes are employed to mimic the cell membrane glycocalyx for constructing an artificial cell membrane glycocalyx nanodecoy that exhibits excellent anti-coronavirus activity against HCoV-OC43, wild-type SARS-CoV-2, Alpha and Delta variant SARS-CoV-2 pseudovirus. In addition, this nanodecoy, loaded with surface sulfate groups as SARS-CoV-2 receptor arrays, can enhance the antiviral capability to virus inactivation through destroying the virus membrane structure and transfer the spike protein to postfusion conformation. Integrating bio-inspired recognition and inactivation of viruses in a single supramolecular entity, the artificial cell membrane nanodecoy opens a new avenue for the development of theranostic antiviral nanosystems, whose mass production is favored due to the facile engineering of sulfated liposomes.

## Highlights

• An antiviral artificial cell membrane glycocalyx nanodecoys with viral inactivation capability was fabricated using sulfated liposomes.
• The artificial cell membrane glycocalyx nanodecoy showed a broad-spectrum anti-coronavirus ability.
• The artificial cell membrane glycocalyx nanodecoy could activate the membrane fusion process of coronavirus, get membrane fusion with the viral membrane and destruct virual structure.
• The membrane fusion process could inactivate viruses by transforming the spike protein of coronavirus from prefusion conformation to postfusion conformation.

## Graphical abstract

**Figure:**
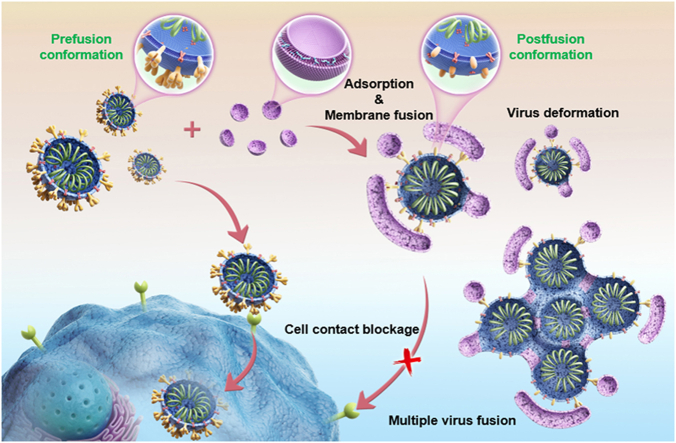

## 1 Introduction

The COVID-19 pandemic caused by SARS-CoV-2 has been raging for years [1,2]. Numerous strategies have been proposed to mitigate this severe worldwide public health problem. Vaccines [3,4], antiviral drugs [5,6], and virus-neutralizing antibodies [7,8] have only partially contained the spread. However, due to the evolution of SARS-CoV-2, the emergence of a multitude of viral variants has greatly restricted the performance of these treatments [[9], [10], [11]]. It has been found that the effectiveness of BNT162b2 and ChAdOx1 against Delta variant was reduced 10–13% (BNT162b2) and 16% (ChAdOx1) compared to Alpha variant [12]. And there are several articles reported that the mutations of SARS-CoV-2 main protease confer drug resistance to nirmatrelvir [13,14]. Cell membrane nanodecoys are novel antiviral nanoparticles constructed by camouflaging with cell membranes, which could capture the target virus according to the antigens inherited from the cell surface [[15], [16], [17]]. The cell membrane nanodecoys have similar surface as the susceptible cells. If the virus can bind with the receptor on the cell surface, they can be captured by the nanodecoys. Therefore, the nanodecoys will not elicit high selective pressure and are considered one of the most promising solutions to the problem of viral mutations [18,19]. Up to now, various nanodecoys against SARS-CoV-2 were developed, which showed excellent antiviral ability. However, because these nanodecoys are built using natural cell membranes, it is difficult to put them into mass production [20]. Furthermore, some of the cell membrane nanodecoys can only catch virus particles and cannot destroy their structure to realize virus inactivation [15]. The viral inactivation is a necessity for good antiviral potency, because some examples show that detachment poses a risk for these captured viruses to recover their infectivity following detachment from the nanodecoys [21].

Liposomes are phospholipid vesicles that have been widely employed in biomedical applications such as drug carriers [22]. In addition, because liposomes have a similar structure to that of the biological membrane, they have been widely used to mimic biological membranes. By altering the composition, liposomes could serve as the biomimetic membranes of cells [23], bacteria [24], and viruses [25]. Among them, a large number of studies used liposomes as cell membrane mimics to explore cell membrane-virus interactions and investigate the fusion mechanisms of influenza virus [26], poliovirus [27], hepatitis B virus [28], and SARS-COV-2 [29] with the cell membrane. The utilization of liposomes as artificial cell membrane nanodecoys for antiviral therapeutics potentiates large-scale production but has yet to be examined.

Heparan sulfate proteoglycan (HSPG) is a highly sulfated proteoglycan located on the cell membrane. Many types of viruses, including the coronavirus, begin the invasion process by attaching to HSPGs [30]. These viruses could multivalently combine with the sulfonate groups on HSPGs to help them accumulate on the plasma membrane [31,32]. Based on this process, various HSPG-mimicking antiviral nanoparticles have been developed. These nanoparticles can effectively bind with viruses, which passivate the viruses by blocking the interactions with the naturally occurring HSPGs of the cell surface, thereby inhibiting viral infection [33,34]. At present, many antiviral drugs that block the interaction between virus and HSPG has been reported, such as gold nanoparticle, cyclodextrin, lactoferrin, etc., which have shown a great antiviral ability [35,36].

Because HSPG is an attachment factor of many viruses, HSPG-mimicking nanoparticles show a broad-spectrum antiviral ability, which could effectively neutralize multiple viruses including HIV, HSV, RSV, DENV, SARS-CoV-2, etc [21,37]. Unfortunately, most of the HSPG-mimicking virus neutralization strategies can only temporarily block viral interaction with cells, and the viral infection effect is retained [38,39]. Upon disengagement from HSPG-mimicking nanoparticles under certain conditions such as dilution, these viruses can infect cells again. Harnessing HSPG-mimics as molecular radars of the coronavirus, we sought to construct a sulfated liposome-based nanodecoy that could permanently inactivate the virus.

**Fig. 1:**
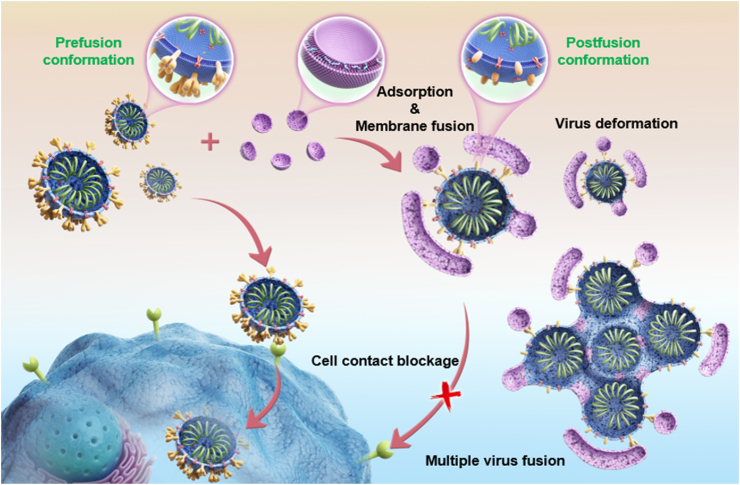
The illustration shows the antiviral mechanism of artificial cell membrane glycocalyx nanodecoys. SARS-CoV-2 infects cells by binding with receptors on the cell membrane. The artificial cell membrane glycocalyx nanodecoys could prevent SARS-CoV-2 from contacting cell membrane receptors by binding with the spike proteins of SARS-CoV-2. Due to the likelihood of membrane fusion, the artificial cell membrane glycocalyx nanodecoys adsorbed on the virus surface could fuse with the viral envelope and other nanodecoys, leading to a series of fusion events and the eventual virus inactivation.

In this research, we fabricated a small-sized HSPG-loaded-cell-membrane-mimicking nanodecoy using an artificial cell membrane glycocalyx based on sulfated liposomes. Two U.S. Food and Drug Administration (FDA) approved excipients, hydrogenated soybean phosphatidylcholine (HSPC) and cholesteryl sodium sulfate (CS) were used to prepare the nanodecoy. The nanodecoys could bind to the spike proteins of the coronavirus due to their sulfate groups and block the interaction between the virus and the cell. More importantly, the cell-membrane-like structure allows the nanodecoys to fuse with the viral envelope and neighboring nanodecoys, enabling them to inactivate the virus by deforming or shielding the virus particles (Fig. 1). We wish this artificial cell membrane glycocalyx nanodecoy could alleviate the impact of SARS-CoV-2 viral mutations by enabling large-scale production and furnishing virus inactivation ability for highly potent prophylaxis of SARS-CoV-2.

## 2 Results and discussion

### 2.1 Physicochemical properties of cell membrane glycocalyx mimicking nanodecoys

**Fig. 2:**
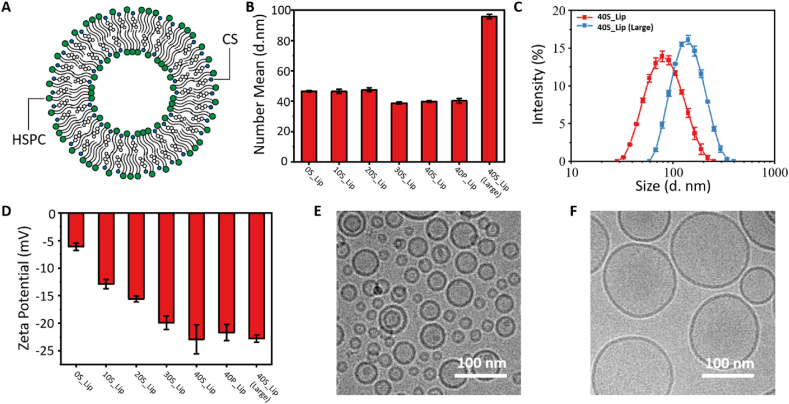
Characterization of artificial cell membrane glycocalyx nanodecoys. (A) The cartoon represents the structure of an artificial cell membrane glycocalyx nanodecoy. (B) The number-average hydrodynamic diameter of artificial cell membrane glycocalyx nanodecoys with different compositions and sizes in PBS at a pH of 7.4 by dynamic light scattering (DLS). (C) The particle size distribution of 40S_Lip and 40S_Lip(Large) in PBS at a pH of 7.4. (D) The zeta-potential of artificial cell membrane glycocalyx nanodecoys with different compositions and sizes in PBS at a pH of 7.4. (E-F) The morphology of (E) 40S_Lip and (F) 40S_Lip(Large) in PBS at a pH of 7.4 by Cryo-TEM.

In this research, HSPC and CS were used to prepare sulfated liposome-based cell membrane glycocalyx mimicking nanodecoys. HSPC served as the starting lipid to construct the basic structure. CS could improve the stability of the nanodecoys by filling the gap between HSPC molecules. The sulfate groups on CS could endow liposomes with HSPG-mimicking characteristics. Since too much CS would affect the stability of the nanodecoys, we prepared four small-sized nanodecoys (denoted as 0S_Lip, 10S_Lip, 20S_Lip, 30S_Lip, and 40S_Lip) with CS proportions from 0 to 40% and one large-sized nanodecoy 40S_Lip(Large) with the same lipid proportion as 40S_Lip (Fig. 2A). The nanodecoys were prepared using a classical filming-rehydration method, and the compositions of the nanodecoys are listed in Table 1. Two different methods were applied to control the size of the nanodecoy. The small-sized nanodecoy was prepared by sonication, which could put extremely high energy into the lipid dispersion and render the nanodecoys with an ultra small size (Fig. S1). The large-sized nanodecoy was prepared by extrusion through a 100 nm polycarborn membrane.

**Table 1:** The physicochemical properties of nanodecoys.

Name	Composition in mole fraction	Size by number (d, nm)	Polydispersity index (PDI)	ζ-potential (mV)
	HSPC/CS/Cholesterol			
0S_Lip	0.80/0.00/0.20	46.64 ± 0.56	0.239	−6.13 ± 0.67
10S_Lip	0.70/0.10/0.20	46.54 ± 1.31	0.152	−12.9 ± 0.83
20S_Lip	0.60/0.20/0.20	47.49 ± 1.27	0.151	−15.6 ± 0.52
30S_Lip	0.50/0.30/0.20	38.81 ± 0.78	0.187	−19.9 ± 1.21
40S_Lip	0.40/0.40/0.20	39.76 ± 0.60	0.270	−22.9 ± 2.63
40S_Lip (Large)	0.40/0.40/0.20	95.82 ± 1.43	0.106	−22.8 ± 0.65

The physicochemical properties of these nanodecoys with different sizes and compositions were characterized at first. Dynamic light scattering (DLS) was used to study the hydrodynamic size of these liposomes. As shown in Fig. 2B and C, the number-average hydrodynamic diameters of the small-sized nanodecoys were about 40 nm and were independent of the lipid proportions. The hydrodynamic diameter of the large-sized nanodecoys was about 100 nm. Then, we studied the surface charge of these liposomes. According to Fig. 2D, the zeta-potential of the nanodecoys decreased with the increase of the proportion of CS. As the proportion of CS increased, the zeta-potential of liposomes decreased from −6.13 ± 0.67 mV to −22.90 ± 2.63 mV, which might be attributed to the strong negative charge of CS. In addition, this result also proved that CS was successfully assembled in the nanodecoys. The morphology of these liposomes was spheroidal, as observed by cryogenic transmission electron microscopy (cryo-TEM). The diameters of the small-sized liposomes ranged from about 20 nm to 90 nm and that of the large-sized liposomes ranged from 80 nm to 200 nm (Fig. 2E–F, S2), which was consistent with the DLS results. In addition, here we also used multi-angle dynamic light scattering (MADLS) to study the particle concentration of these nanodecoys (Fig. S3). We found that for 0.1 mg mL-1 nanodecoy, the particle concentration of small-sized nanodecoy was about 1E+12 particles mL-1 and the large-sized nanodecoy was about 1E+11 particles mL-1, which was about 10 times lower than small-sized nanodecoy.

### 2.2 Antiviral ability of nanodecoys against lentivirus

The antiviral ability of these nanodecoys was then investigated using a green fluorescent protein (GFP) gene-loaded VSV-G lentivirus (LV) as a model virus. Lentivirus is a viral vector developed from HIV-1, which has a similar infection mechanism as HIV-1. Both LV and HIV-1 rely on HSPGs to adhere to the surface of cells. However, LV cannot replicate and shows excellent biosafety, which justifies its wide usage as a model virus. If the virus infects the cell, the cell would express GFP and emit green fluorescence. Therefore, the infection rate of the viruses can be studied with fluorescence microscopy or flow cytometry.

**Fig. 3:**
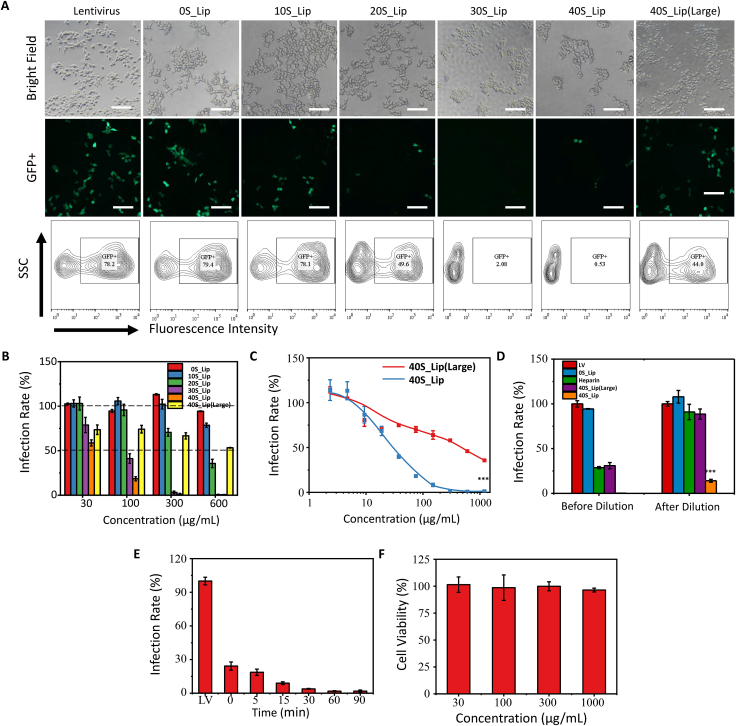
Characterization of the antiviral ability and cytotoxicity of nanodecoys. (A) The bright-field (top row), the fluorescence (medium row) images, and the flow cytometry figure (bottom row) of HEK-293T cells infected by the lentivirus incubated with 300 μg mL-1 nanodecoys at 37 °C for 1 h. Scale bar: 200 μm. (B) The infection rate of lentivirus after incubating with different concentrations of nanodecoys at 37 °C for 1 h. (C) The infection rate curve of lentivirus incubated with different concentrations of 40S_Lip and 40S_Lip(Large). (D) The infection rate of the lentivirus incubated with 900 μg mL-1 heparin, 40S_Lip(Large), and 40S_Lip for 1 h at 37 °C before or after dilution. (E) The infection rate of lentivirus incubated with 300 μg mL-1 40S_Lip at different times. (F) The viability of NIH-3T3 cells after incubation with different concentrations of 40S_Lip. ***p < 0.001 by *t*-test.

Firstly, the effects of composition and size of the nanodecoys on the anti-infection ability were studied. After incubating LV with different nanodecoys for a pre-determined time, LV was then incubated with HEK-293T cells to study the infectivity of LV by fluorescence microscopy and flow cytometry. As shown in Fig. 3A, the infectivity of LV remained almost unchanged after co-incubation with 300 μg mL-1 of 0S_Lip and 10S_Lip. In contrast, if LV was incubated with 300 μg mL-1 of 20S_Lip, 30S_Lip, or 40S_Lip, the infectivity of LV decreased with the increase of CS proportion in the nanodecoys. The anti-infective ability of the nanodecoys was also concentration-dependent. The increase of the nanodecoy concentration would reduce the infectivity of the lentivirus (Fig. 3B). The half-maximal effective concentration (EC50) values of the 0S_Lip, 10S_Lip, 20S_Lip, and 40S_Lip(Large) were above 600 μg mL-1.30S_Lip, and 40S_Lip were 72.48 μg mL-1, and 34.99 μg mL-1, respectively. These results show that the antiviral ability of the nanodecoys increased with the increase of CS proportion, which imply the critical role of CS in inhibiting viral infection. The virus might interact with CS by multivalent binding. It has been reported that with bivalent interactions, the affinity of the virus to the attachment factors increases 250-fold when compared to monovalent binding [40,41]. With the increase of CS proportion in the nanodecoy, the number of binding sites on the nanodecoy also increases, which could significantly strengthen the binding between the virus and nanodecoy, leading to enhanced antiviral ability of the nanodecoys.

In addition, we studied the influence of particle size on the antiviral ability of the nanodecoys by comparing the antiviral ability of the small-sized nanodecoy 40S_Lip and large-sized nanodecoy 40S_Lip(Large). Although 40S_Lip and 40S_Lip(Large) had the same composition, the antiviral ability of 40S_Lip(Large) was far worse than 40S_Lip (Fig. 3C). The EC50 of 40S_Lip(Large) was above 600 μg mL-1, which was much higher than that of 40S_Lip (34.99 μg mL-1). This may be due to the relatively small specific surface area of the large-sized nanodecoy, which makes 40S_Lip(Large) much harder to adhere to the virus, resulting in the relatively poor antiviral ability of 40S_Lip(Large). Therefore, small particle size is important in maintaining the high antiviral ability of nanodecoys. 40S_Lip with the best antiviral performance was used in the following research.

As discussed above, HSPG and HSPG-mimicking nanoparticles can neutralize viruses via the interaction between sulfonate groups of HSPG and viruses, which can prevent viruses from attaching to cells [32]. Unfortunately, the neutralized viruses might not be inactivated [21]. Once HSPG or HSPG-mimicking nanoparticles are detached from the surface of the virus, the infectivity of the virus would recover, which implies the potential risk of this antiviral strategy. It will be much more promising if the nanoparticles could disrupt the structure of and thereby inactivate the virus. In this case, the viruses will not be able to infect cells anymore. It is very important to investigate if the nanodecoy can inactivate viruses. Heparin is a natural sulfated polymer, which was reported to inhibit virus infection by competitive binding with viruses [42,43]. Heparin was used as a control to study the virus inactivation ability of the nanodecoy. If the nanodecoys are not able to inactivate viruses, they might detach from the virus surface after dilution, leading to the recovery of infection ability.

### 2.3 Inactivation of lentivirus

Here, the LV and HEK-293T cells were also used to study if the nanodecoy could inactivate viruses. We firstly incubated the LV with the nanodecoy or heparin for 1 h and then diluted the solution 1000 times using cell culture medium to study the infection rate change of the treated virus. The results were normalized to the virus only group which servered as a control. As shown in Fig. 3D, the infection ability of the LV incubated with heparin was greatly recovered after extensive dilution. The heparin decreased the infection rate to 35.70 ± 0.25% before dilution, but the infection rate recovered to 94.93 ± 18.60% after dilution, which indicated that heparin could not inactivate viruses, giving rise to regaining of the infection ability upon heparin detachment. For the LV incubated with 40S_Lip, the infection rate only increased from 1.26 ± 0.89% to 2.66 ± 0.49% after extensive dilution, which remained a very low infection rate. This result means 40S_Lip could not only neutralize the virus but also inactivate the virus. Meanwhile, the infection rate of the virus incubated with 40S_Lip(Large) was also significantly increased after extensive dilution, which might be ascribed to the relatively low binding force between 40S_Lip (Large) and LV.

The incubation time dependency of the antiviral activity of the nanodecoys was also studied. After incubating LV with 300 μg mL-1 40S_Lip at 37 °C for different time intervals, cells were added to study the infectivity of LV. The infection rate of LV was then calculated by using flow cytometry. As shown in Fig. 3E, if cells were added into LV solution immediately after the addition of 40S_Lip (without pre-incubation), the infection rate of the virus dramatically decreased to 24.21 ± 3.65%. Moreover, the infection rate of LV decreased with the extension of pre-incubation time. After LV was pre-incubated with 40S_Lip for 30 min, the infection rate of LV decreased to as low as 3.89 ± 0.38%, which indicated that 40S_Lip could completely block the infection of LV. This result showed that the nanodecoys could reduce viral infectivity in a very short time, which endows the nanodecoys with great potential in preventing virus spread.

At last, the cytotoxicity of these nanodecoys were characterized. After incubating bronchial epithelial cells, NIH-3T3, and HEK-293T cells with different concentrations of nanodecoys, the cell viability of the cells was studied by using cell counting kit-8. According to Fig. 3F and S4, even at the concentration of 2000 μg mL-1, these nanodecoys did not cause a decrease in cell viability. Notably, 40S_Lip almost completely suppressed virus infection at this concentration, indicating the safety of 40S_Lip for antiviral usage.

After preliminarily studying the antiviral effect, we further investigated whether these nanodecoys achieve antiviral function through HSPG biomimetic strategy. It is known that the spike proteins on coronavirus interact intensely with the sulfonate groups of HSPGs by multivalent binding [30,44]. Therefore, heparin-like sulfated or sulfonated supramolecular assemblies such as the nanodecoys might strongly bind with the spike proteins on LV by multivalent binding, which could block the interaction between LV and HEK-293T cells, thus inhibiting viral infection. A phosphorylated liposome 40P_Lip was prepared as a negative control using hexadecyl hydrogen phosphate and HSPC, which did not have sulfate groups but showed a similar surface charge with 40S_Lip. Compared with 40S_Lip, the antiviral ability of 40P_Lip was significantly lower (Fig. S5). LV could no longer infect cells after incubation with 40S_Lip but retained its original infectious ability after incubating with 40P_Lip. This result reveals that the excellent antiviral ability of 40S_Lip might be ascribed to the existence of sulfate groups on the liposomes. In addition, the non-HSPG-dependent virus, adeno-associated virus 5 (AAV5) and vesicular stomatitis virus (VSV), was used as a negative control to study the mechanism of the antiviral ability of these nanodecoys. If the nanodecoys neutralize the virus through HSPG mimicking strategy, they should not be able to neutralize the non-HSPG-dependent virus. According to the flow cytometry results and the fluorescence microscopy images (Figs. S6 and S7), AAV5 and VSV still maintained very high infectivity even if they were incubated with 1200 μg mL-1 40S_Lip, which indicated that these nanodecoys were not able to neutralize the non-HSPG-dependent virus. These results show that the nanodecoys realized their antiviral function through the HSPG-mimicking strategy.

We also used the time-of-addition assay to investigate whether the antiviral ability of these nanodecoy came from inhibiting viruses or regulating cells. As shown in Fig. S8, when the cells were pre-incubated with 40S_Lip for 1 h and then washed with PBS before the addition of lentivirus (Pretreatment cells), they could still be infected by the virus. However, if we first incubated the virus with the nanodecoy for 1 h and then added the mixed solution to cells (Pretreatment Virus); or added the virus and the nanodecoy to the cell at the same time (During Infection), the infectivity of the virus was significantly reduced. Compared with the During Infection group, a much lower infection rate was observed in the Pretreatment Virus group. This phenomenon shows that the nanodecoy achieved its antiviral function by acting on the virus. The liposomes in the Pretreatment Virus group had the longest time to interact with the virus so that the virus could be fully blocked by the liposomes, leading to the best antiviral effect. However, because the liposomes in the Pretreatment Cell group only incubated with cells but did not contact the virus, all viruses were not blocked, which resulted in the highest infection rate.

### 2.4 Suppression of SARs-CoV-2 infections using artificial cell membrane glycocalyx nanodecoy

**Fig. 4:**
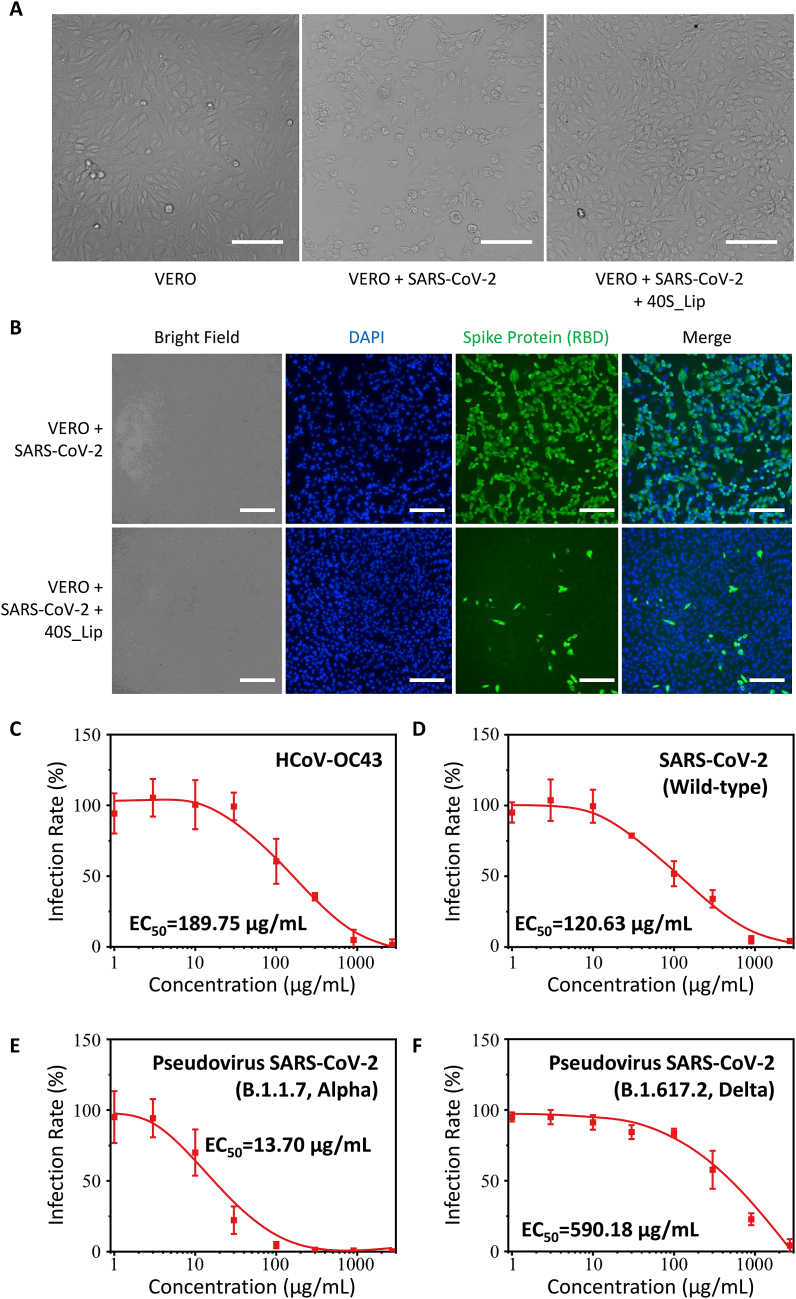
The anti-coronavirus ability of nanodecoys. (A) The micrograph of Vero cells after incubation with single SARS-CoV-2 or both SARS-CoV-2 and 900 μg mL-1 40S_Lip to reveal the cytopathic effect (CPE). Scale bar: 100 μm. (B) The fluorescence image of VERO cells after incubation with single SARS-CoV-2 or both SARS-CoV-2 and 900 μg mL-1 40S_Lip. Cell nuclei were labeled with DAPI (blue) and the spike protein S1 RBD of SARS-CoV-2 was labeled with Alexa Fluor 488 (Green). Scale bar: 100 μm. (C-D) The infection rate curve of coronavirus (C) HCoV-OC43, (D) wild-type SARS-CoV-2 after incubation with 40S_Lip of different concentrations for 1 h at 37 °C. (E-F) The infection rate curve of pseudovirus (E) SARS-CoV-2 Alpha variant pseudovirus, and (F) SARS-CoV-2 Delta variant pseudovirus after incubation with 40S_Lip of different concentrations for 1 h at 37 °C.

Following characterizations of the antiviral ability and preliminarily understanding the antiviral mechanism of the nanodecoy, we further studied whether these nanodecoys could suppress the infection of SARS-CoV-2. Here, we both used wide-type SARS-CoV-2 and SARS-CoV-2 pseudovirus to do this study. The pseudovirus was a replication-defective virus based on HIV, whose surface protein and the attachment and entry process were the same as the SARS-CoV-2 but the internal gene was GFP. The infection of pseudovirus would also cause cells to emit green fluorescence. Firstly, the wild-type SARS-CoV-2 was used to investigate the antiviral ability of the nanodecoys. Because SARS-CoV-2 could induce cytopathic effects (CPEs), we initially used Vero cells to study if the nanodecoys could suppress the CPE. By incubating Vero cells with SARS-CoV-2 or SARS-CoV-2 with 0S_Lip or 40P_Lip, the morphology of Vero cells changed remarkably (Fig. 4A, S9), which indicated that SARS-CoV-2 could induce CPE and the deformation of cells. However, when the Vero cells were incubated with SARS-CoV-2 and 900 μg mL-1 40S_Lip, the cell morphology was barely changed, suggesting the nanodecoys could suppress the CPE induced by SARS-CoV-2. In addition, we also used the immunofluorescence method to figure out if the nanodecoys could prevent cells from being infected by the SARS-CoV-2. The Alexa Fluor488 labeled antibody was used to label the receptor-binding domain (RBD) of the SARS-CoV-2 spike protein. According to Fig. 4B, after incubating the Vero cell with the SARS-CoV-2 for 48 h, almost all cells expressed spike proteins, which means that these cells were infected by the SARS-CoV-2. But for the Vero cells incubated with SARS-CoV-2 and 40S_Lip, the number of the spike-protein-expressing cells dramatically decreased. This result proves that the nanodecoys could prevent the SARS-CoV-2 from invading the cells. Collectively, these two results confirm that 40S_Lip could reduce the infection rate of the lentivirus and protect cells from SARS-CoV-2.

As mentioned before, since HSPG is a highly conserved viral attachment factor, nanoparticles constructed based on the HSPG-mimicking strategy usually possess the broad-spectrum antiviral ability. In light of this characteristic, the sulfated nanodecoy may have the potential to neutralize multiple variants of the coronavirus. To study whether the nanodecoys could treat multiple variants of coronavirus, the antiviral activity of 40S_Lip was tested using 4 variants of the coronavirus, including two kinds of live coronaviruses and two kinds of SARS-CoV-2 pseudovirus (Fig. 4C–F). As shown in Fig. 4C and D, 40S_Lip showed a similar antiviral ability to HCoV-OC43 and wild-type SARS-CoV-2. The EC50 values of 40S_Lip against HCoV-OC43 and wild-type SARS-CoV-2 were 189.7 μg mL-1 and 120.6 μg mL-1, respectively. It is worth noting that HCoV-OC43 belongs to the subpopulation Embecovirus and SARS-CoV-2 belongs to the subpopulation Sarbecovirus, which means that these two kinds of coronavirus have very different spike protein structures. Therefore, 40S_Lip can bind to the surface of different coronavirus varient to block the invasion process. We also stuided the titer of SARS-CoV-2 incubated with 900 μg mL-1 40S_Lip using TCID50 assay, which was found that the TCID50 of SARS-CoV-2 decreased from 1000 TCID50 mL-1 to 2.375 TCID50 mL-1. Besides, we also studied the antiviral ability of 40S_Lip to two kinds of SARS-CoV-2 pseudovirus of different variants. According to Fig. 4E and F, the EC50 values of 40S_Lip to Alpha variant and Delta variant SARS-CoV-2 pseudovirus were 13.70 μg mL-1 and 590.1 μg mL-1, respectively. Meanwhile, we also studied if the 40S_Lip could treat high concentration virus (Fig. S10.). Here by treating wide-type SARS-CoV-2 of different concentration with 900 μg mL-1 40S_Lip, none of the virus could infect the Vero cell. These results indicate that this nanodecoy could inhibit cell infections cause by multiple coronaviruses.

### 2.5 Anti-coronavirus mechanism of artificial cell membrane glycocalyx nanodecoy

**Fig. 5:**
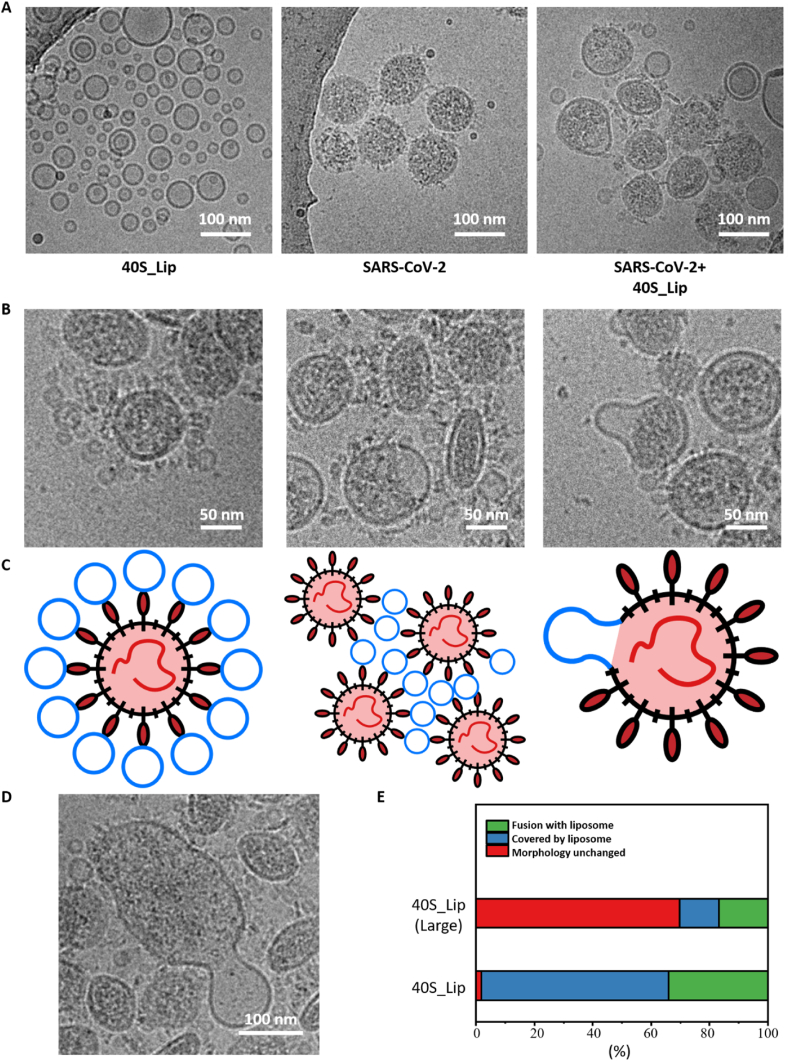
The interaction between nanodecoys and SARS-CoV-2 according to cryo-TEM. (A) The cryo-TEM image of 40S_Lip, SARS-CoV-2, and the SARS-CoV-2 incubated with 40S_Lip. Scale bar: 100 nm. (B) The cryo-TEM image and (C) the respective cartoons showcase different interaction modes between SARS-CoV-2 and 40S_Lip. Scale bar: 50 nm. (D) The cryo-TEM image of the fused virus after incubation with 40S_Lip. Scale bar: 100 nm. (E) Statistics on the proportion of interaction modes between SARS-CoV-2 and 40S_Lip or 40S_Lip(Large).

To further explore the anti-coronavirus mechanism of this nanodecoy, cryo-TEM was used to characterize how the nanodecoys interacted with SARS-CoV-2 viruses. According to the cryo-TEM image in Fig. 5A, 40S_Lip had a very strong interaction with SARS-CoV-2, it has been found that each virus have 7–20 liposomes attached on their surface. There were multiple interaction modes between 40S_Lip and SARS-CoV-2, including entrapment, aggregation, and membrane fusion (Fig. 5B and C). In the entrapment mode, the virus was covered by the nanodecoys due to the strong binding between sulfate groups on the nanodecoys and spike proteins on viruses. The viruses covered by the nanodecoys would not be able to interact with cells, leading to virus neutralization by the nanodecoys. In the aggregation mode, the nanodecoys could mediate SARS-CoV-2 to aggregate. This was mainly because a nanodecoy could bind with multiple viruses and a virus could interact with multiple nanodecoys, which made the nanodecoy serve as a crosslinking agent for the aggregation of viruses. More interestingly, the nanodecoys could fuse with the envelope of SARS-CoV-2, leading to the occurrence of membrane fusion. The liposomal nanodecoys have a similar structure to the cell membrane and the viral envelope. After the nanodecoy binds to the surface of the virus, there is a high probability of membrane fusion between the nanodecoy and the viral envelope. The membrane fusion would cause the virus deformation, which would disrupt the virus structure and eventually causes viral inactivation [45,46]. It is important to note that these three interaction modes are not mutually exclusive. They may occur simultaneously and eventually result in multiple viruses fusing into a large, malformed fusion body (Fig. 5D). In addition, the interaction between SARS-CoV-2 and the 0S_Lip and 40S_Lip(Large) was also studied. Compared with 40S_Lip, both the 0S_Lip or 40S_Lip(Large) showed a much weaker interaction with SARS-CoV-2. Few nanodecoys were observed to cover the surface of the virus, and most viruses retained their original morphology (Fig. S11).

Taken further, we studied the statistics on the proportion of these three interaction modes. As shown in Fig. 5E, for SARS-CoV-2 incubated with 40S_Lip(Large), the majority of SARS-CoV-2 maintained their original morphology, which implies that these viruses were still able to infect cells. On the contrary, most of the SARS-CoV-2 incubated with 40S_Lip showed a morphology change. About 64.22% of the viruses were covered by nanodecoys. 33.94% of the viruses showed membrane fusion, and only 1.835% of the viruses could keep their original morphology. This result reveals that 40S_Lip had a very strong interaction with the viruses, which could not only neutralize viruses by entrapment and aggregation but also inactivate viruses by membrane fusion.

**Fig. 6:**
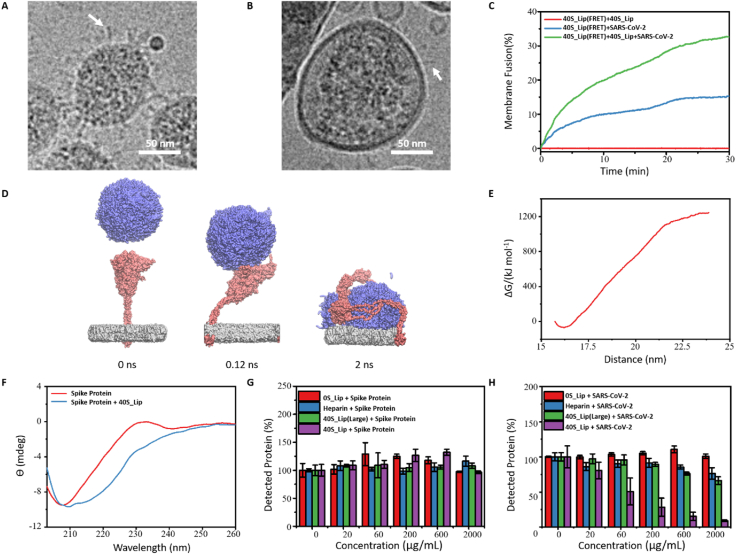
Nanodecoy membrane fusion causes virus block. The cryo-TEM image of (A) SARS-CoV-2 or (B) SARS-CoV-2 incubated with 40S_Lip. The arrow pointing the spike protein. Scale bar: 50 nm. (C) The membrane fusion profile of the FRET lipid labeled nanodecoy 40S_Lip(FRET) incubated with no FRET lipid labeled nanodecoy 40S_Lip, SARS-CoV-2 pseudovirus, or 40S_Lip and SARS-CoV-2 pseudovirus. (D) The MD simulation result of the spike protein incubated with 40S_Lip for 0, 0.12, and 2 ns. (E) The potential of mean force curves for 40S_Lip and S protein. (F) The CD spectrum of the native spike protein or when incubated with 40S_Lip. (G) The percentage of the detected free spike protein after incubation with heparin, 40S_Lip(Large), or 40S_Lip. (H) The percentage of the detected spike protein on the SARS-CoV-2 pseudovirus after incubation with heparin, 40S_Lip(Large), or 40S_Lip.

According to the cryo-TEM images, it was very interesting to find that the shape of the spike proteins on the surface of the virus particles changed to a needle-shaped form after incubating with 40S_Lip, and some of the virus particles showed a double bilayered structure on the surface (Fig. 6A–B, S12). This phenomenon might be ascribed to the occurrence of membrane fusion. 40S_Lip had relatively high surface energy due to its small size and tend to fuse to lower the surface energy. However, because of the strong electrostatic repulsion, 40S_Lip particles were not able to get into contact with each other, which resulted in the absence of membrane fusion. However, after adhering to the surface of the viruses, 40S_Lip could interact with the virus and neighboring liposomes, leading to the occurrence of membrane fusion. Therefore, the liposomes on the surface of the virus would fuse with each other, which might lead to the formation of a giant liposome on the viral surface to wrap the virus particle eventually. Meanwhile, some liposomes would fuse with virus particles to disrupt the virus structure. This membrane fusion would lead to the spike protein change from prefusion conformation to postfusion conformation, which made the spike protein showed a niddle-liked shape and let the virus lose infectivity [47,48]. Both of these two membrane fusion modes might explain the change of the SARS-CoV-2 in the cryo-TEM images. As such, the cascade of bio-recognition in combination with membrane fusion events triggered by the introduction of nanodecoys may amplify the antiviral activity.

To observe virus-assisted membrane fusion, the fluorescence resonance energy transfer (FRET) assay was applied to study if the adhesion of nanodecoys to the virus surface would promote membrane fusion among nanodecoys. Two phosphoethanolamine (PE) conjugated FRET probes, NBD-PE and Rhodamine-PE, were used to prepare FRET probe labeled nanodecoys (40S_Lip(FRET)). The emission wavelength of NBD is 539 nm, which is very close to the excitation wavelength of Rhodamine B (554 nm). For the nanodecoy labeled with both NBD-PE and Rho-PE, the NBD fluorescence attenuated because of FRET to Rhodamine B. Once the membrane fusion occurred, the NBD signal increased because of the larger distance between NBD-PE and Rho-PE. Based on this strategy, we incubated 40S_Lip(FRET) with non-labeled 40S_Lip, wild-type SARS-CoV-2 pseudovirus, or both 40S_Lip and wild-type SARS-CoV-2 pseudovirus to figure out if the adhesion of 40S_Lip on the virus would promote membrane fusion. As shown in Fig. 6C, membrane fusion was not observed after incubating 40S_Lip(FRET) with 40S_Lip since the fluorescent emission of NBD was not detected, which implies that 40S_Lip would not fuse with each other in the absence of viruses. After incubating 40S_Lip(FRET) with SARS-CoV-2 pseudovirus, the emitted fluorescence of NBD significantly increased. After 30 min incubation, about 15.30% lipid mixing occurred, indicating that 40S_Lip could fuse with the envelope of the virus, which was consistent with cryo-TEM results. After 40S_Lip(FRET) was co-incubated with SARS-CoV-2 pseudovirus and 40S_Lip, the strongest fluorescence of NBD was observed. About 32.62% lipid mixed after 30 min incubation, indicating the occurrence of the most obvious membrane fusion phenomenon. Therefore, when 40S_lip adsorbed on the surface of the virus, they would not only fuse with the viral envelope but also fuse with neighboring nanodecoys, which suggests that the adsorption of 40S_lip on the virus surface could promote membrane fusion between nanodecoys. In contrast, by incubating the 0S_Lip or 40P_Lip with virus, barely FRET could be observed (Fig. S13), which proved that with low virus binding affinity, it's hard to induce the membrane fusion of the liposome.

Then, we studied the binding force between the 40S_Lip and spike protein of the virus. The molecular dynamics (MD) simulation and the circular dichroism (CD) spectrum analysis were performed. The MD result showed that as the 40S_Lip approached the virus, the barycenter of 40S_Lip and spike protein obviously shifted. After 2 ns, 40S_Lip even caused spike protein to flat on the virus surface and lead to the change of the secondary structure of spike protein (Fig. 6D, S14). The potential of mean force calculations showed that the binding energy between 40S_Lip and spike protein was up to 1311.91 kJ mol-1, which indicates that the 40S_Lip and spike protein had a very strong interaction (Fig. 6E).

In the end, to unravel whether the membrane fusion could transfer the spike protein to postfusion conformation, we characterized the change of CD spectrum of spike protein before and after incubating with 40S_Lip to study the changes in the secondary structure of proteins caused by 40S_Lip. According to Fig. 6F, the CD spectrum of spike protein significantly changed after incubating with 40S_Lip. The peak at 208 nm shifted to 211 nm, which means the secondary structure changed. These results verify 40S_Lip had a very strong interaction with spike protein, indicating that 40S_Lip has the ability to tightly adhere to the surface of the SARS-CoV-2. In addition, the sandwich enzyme-linked immunosorbent assay (ELISA) was used to study whether the RBD of spike protein on the virus surface could be detected after incubating SARS-CoV-2 pseudovirus with 40S_Lip. According to Fig. 6G, the free spike protein RBD could be detected by ELISA after incubating with heparin or nanodecoys. It is may because without membrane fusion, the nanodecoy would not able to transform the spike protein RBD to postfusion conformation. Then, the SARS-CoV-2 pseudovirus was incubated with different concentrations of nanodecoys (Fig. 6H). When the concentration of heparin was as high as 600 μg mL-1, 40S_Lip(Large) showed excellent antiviral ability. However, more than 85.61 ± 2.82% or 76.18 ± 2.02% spike protein on the SARS-CoV-2 pseudovirus still could be detected after incubating with 600 μg mL-1 heparin and 40S_Lip(Large). But for 40S_Lip, the amount of detected spike protein declined with the increase of the nanodecoy concentration. Only 15.28 ± 5.93% of spike protein could be detected after 600 μg/mL 40S_Lip was incubated with SARS-CoV-2 pseudovirus. We speculated the potential reason of this phenomenon. Heparin and 40S_Lip(Large) might not induce the transformation of spike protein. However, 40S_Lip might transfer the conformation of spike protein on SARS-CoV-2. The main reason for this phenomenon might be attributed to the membrane fusion of 40S_Lip with the viral envelope and other 40S_Lip. This process activate the membrane fusion mechanism of spike protein, which made the spike protein transfer to the postfusion conformation. And the membrane fusion also caused the spike protein on the virus surface be covered by the nanodecoy membrane, which caused a permanently shield of the spike protein. Heparin had no membrane structure and 40S_Lip(Large) had a weak interaction with the virus, which rendered heparin and 40S_Lip(Large) unable to fuse with the viral envelope, leading to the detection of the spike protein on SARS-CoV-2 by ELISA.

## 3 Conclusions

In summary, sulfated liposome-based cell membrane glycocalyx mimicking nanodecoys were facilely prepared using two FDA-approved excipients, HSPC and CS. With the incorporated HSPGs as molecular radar arrays for sensing of coronaviruses, we demonstrated that the cell membrane glycocalyx mimicking nanodecoys could effectively reduce the infection rate of four variants of coronavirus, encompassing the wild-type SARS-CoV-2, HCoV-OC43, Alpha variant SARS-CoV-2 pseudovirus, and Delta variant SARS-CoV-2 pseudovirus, indicating the nanodecoy could treat multiple variants of coronavirus. More importantly, the nanodecoys could not only neutralize the virus by entrapping the virus particles but also inactivate the viruses by fusing with the viral envelope, which could cause the deformation of virus particle and transfer the spike protein to postfusion conformation. To the best of our knowledge, this is the first-time report of virus inactivation by membrane fusion with nanotechnology-enabled sulfated liposomes. Such an artificial cell membrane glycocalyx nanodecoy provides an approach to alleviating the problem of strong mutability of the coronavirus and improving the scalability of cell-membrane nanodecoys production, which can be leveraged for the highly potent prophylaxis of SARS-CoV-2. In addition, because the HSPG is a common target for many kinds of virus, this kind of nanodecoy could be expected to inhibit other viruses which similarly use HSPG co-factors for viral entry. It also lays the groundwork for researching new-concept theranostic antiviral nanosystems with a two-in-one working mechanism combining bio-recognition and virus inactivation.

## 4 Materials and methods

### 4.1 Materials

Hydrogenated soybean phosphatidylcholine (HSPC), cholesteryl sodium sulfate (CS), and cholesterol (Cho) were purchased from AVT Pharmaceutical Tech Co., Ltd. (Shanghai, China). NBD-PE, Rhodamine-DHPE, and Alexa Fluor 488-conjugated Goat anti-rabbit IgG were purchased from Invitrogen Co., Ltd. (Shanghai, China). Potassium hexadecyl hydrogen phosphate was purchased from Bide Pharmatech Ltd. (Shanghai, China). HEK-293T, NIH-3T3, and CHO-K1 cells were kindly provided by Stem Cell Bank, Chinese Academy of Sciences. Vero cells and HCoV-OC43 coronavirus were purchased from the American Type Culture Collection (ATCC). Wild-type SARS-CoV-2 was isolated from a patient's sputum sample and validated by sequence analysis of the complete genome (hCoV-19/Hangzhou/ZJU-05/2020, GISAID Accession ID: EPI_ISL_415,711). GFP gene-loaded lentivirus, adeno-associated virus 5 (AAV5), Alpha variant SARS-CoV-2 pseudovirus, and Delta variant SARS-CoV-2 pseudovirus were purchased from PackGene Biotech Co., Ltd. (Guangzhou, China). Wild-type SARS-CoV-2 pseudovirus and ACE2-overexpressing HEK-293T cells were purchased from Future Biotherapeutics Co., Ltd. (Suzhou, China). DMEM medium and F-12K medium were purchased from Gibco Co., Ltd. (USA). MEM medium and HEPES buffer were purchased from Sigma-Aldrich Co., Ltd. (USA). Fetal bovine serum (FBS) was purchased from Tianhang Biotechnology Co., Ltd. (Zhejiang, China). The cell counting kit-8 (CCK-8), DAPI, BSA, and 8% paraformaldehyde were purchased from Solarbio Science & Technology Co., Ltd. (Beijing, China). SARS-CoV-2 (2019-nCoV) spike-RBD antibody, rabbit mAb was purchased from SinoBiological Corp., Ltd. (Beijing, China). The nucleic acid extraction kit (magnetic beads), novel coronavirus (2019-nCoV) multiplex real-time PCR (RT-PCR) kit, and human coronavirus (HCoV-OC43) RT-PCR kit were purchased from Liferiver Bio-Tech Corp., Ltd. (Shanghai, China). The 2019-nCoV Spike Protein RBD was purchased from Beyotime Biotechnology co., Ltd. (Shanghai, China). The SARS-CoV-2 spike protein S1 RBD ELISA kit was purchased from Elabscience Biotechnology Co., Ltd. (Wuhan, China). Methanol, chloroform, dimethylsulfoxide, and other reagents were of reagent or AR grade and purchased from Sinopharm Chemical Reagent Co., Ltd. (Shanghai, China). Deionized water was purified by a Millipore water purification system with a resistivity of 18.0 MΩ cm.

### 4.2 Preparation of artificial cell membrane glycocalyx nanodecoys

The cell-membrane-mimicking nanodecoys were prepared according to the filming-rehydration method. A mixed solution made of chloroform and methanol (8:2) was used to prepare HSPC, CS, potassium hexadecyl hydrogen phosphate, and cholesterol solution with a concentration of 10 mM. The prepared lipid solution was loaded in a pear-shaped flask in proportion and aapproximately 20 mL of mixed solution was added subsequently to ensure the lipids were well dissolved. The flask was incubated in a 55 °C water bath for 15 min and then the solution was placed in a rotary evaporator. After rotary evaporation, a lipid film should be formed on the flask wall. Phosphate buffer saline (PBS) was added to the flask and the lipid film would be peeled by hand shaking or using an ultrasonic cleaner.

Small-sized nanodecoys were prepared by sonication. The prepared lipid aqueous solution was transferred to a centrifuge tube, then the solution was sonicated by an ultrasonic cell crusher for 10 min at 40 W. Large-sized nanodecoys were prepared with a mini-extruder (Avanti, USA). assembled according to the manufacturer's instruction, the lipid solution was extruded through a porous polycarbonate (PC) membrane with a pore diameter of 100 nm for 20 cycles.

Characterization of the nanodecoys: The hydrodynamic size and zeta-potential of nanodecoys were studied by using the Zetasizer (Nano-ZA, Malvern, UK). The morphology of the nanodecoys was studied by electron cryo-microscopy (Talos F200C, FEI, USA).

### 4.3 Cell culture

HEK-293T, ACE2-overexpressing HEK-293T, CHO-K1, and NIH-3T3 cells were cultured with a high-glucose DMEM medium. Vero cells were cultured with the MEM medium. All cell culture media were supplemented with 100 U/mL penicillin, 100 μg/mL streptomycin, and 10% fetal bovine serum (FBS). Cells were cultured at 37 °C in a 5% CO2 environment.

### 4.4 Anti-pseudovirus capacity test

HEK-293T cells (for lentivirus), CHO-K1 cells (for AAV5), or ACE2-overexpressing HEK-293T cells (for SARS-CoV-2 pseudovirus) were seeded in a 48-well plate with a density of 20,000 cells per well. After 24 h, the virus solution was diluted to a titer of 10^5^ TU/mL. The nanodecoys or heparin were gradiently diluted using the virus solution and incubated at 37 °C for 1 h. After incubation, the cell media was removed and 200 μL of the virus solution was added to the well (MOI = 1) and cultured overnight. Then, the virus solution was removed and fresh medium was added to the cell for another 24 h culture. In the end, the infection condition of the cell was studied by using a fluorescence microscope or flow cytometer. The infection rate of the virus could be calculated according to the formula: Infection Rate (%)=(GFP positive% (Sample) - GFP positive% (Blank))/(GFP positive% (Virus) - GFP positive% (Blank)) × 100%

### 4.5 Anti-coronavirus test

Vero cells were seeded in 96-well plates one day prior to the test. The nanodecoys were diluted with serum-free MEM medium at 1:3 (v/v). The nanodecoys at different dilutions were mixed thoroughly with SARS-CoV-2 or OC43 virus cultures (200 TCID50) diluted in equal volumes (50 μL+50 μL) in 96-well plates, respectively, and were incubated at 37 °C for 2 h. At the end of incubation, 100 μL of Vero cells (10^5^/ml) resuspended in cell culture medium were added to each well and incubated in a 5% CO2 incubator at 35 °C. After 6 days of virus incubation has elapsed, the antiviral effect was determined by observing the CPE, performing qRT-PCR, and immunofluorescence test to determine the antiviral effect. All experiments involving infectious virus were conducted in a China National Accreditation Service for Conformity Assessment (CNAS) approved biosafety level III laboratory, State Key Laboratory for Diagnosis and Treatment of Infectious Diseases in Zhejiang University.

### 4.6 RT-PCR

Viral genes were detected by using an RT-PCR kit. Cell supernatants were subjected to automatic nucleic acid extraction (Liferiver Biotech) with a magnetic beads-based kit. Then, SARS-CoV-2 or OC43 nucleic acid abundance was measured with RT-PCR according to the manufacturer's instructions.

### 4.7 Immunofluorescence test

The cell supernatant was discarded. The cells were washed with PBS. 80% acetone solution pre-chilled at −20 °C was added to each well and fixed at 4 °C for 20 min. After washing three times with ice-cold with PBS, the cells were blocked with 1% BSA at room temperature for 30 min. After blocking, rabbit anti-SARS-CoV-2 spike antibody diluted 1000 times with 0.1% BSA was added and incubated at 4 °C overnight. The cells were then washed and goat anti-rabbit IgG (Alexa Fluor488®) diluted 1500 times with 0.1% BSA was added and incubated at room temperature for 1 h in the dark. After washing, DAPI diluted to 0.5 μg/mL was added and stained the nuclei for 5 min at room temperature. Finally, the cells were imaged under an immunofluorescence microscope (Bio-Rad).

### 4.8 Cytotoxicity of nanodecoys

NIH-3T3 cells were seeded in a 96-well plate for 24 h. Then, the nanodecoy with different concentrations was added to the cell. After 24 h incubation, cell viability was studied by using CCK-8. The cell viability was calculated according to the formula: Cell viability (%) = [OD (Sample)-OD (Blank)]/[OD (Control)-OD (Blank)] × 100%

### 4.9 Cryo-TEM studies of the interaction between nanodecoys and virus

Five microliter 10^12^ TU mL-1 SARS-CoV-2 was pre-incubated with 5 μL 10 mg mL-1 nanodecoy at 37 °C for 1 h. Two microliter of the cocktail was loaded on a glow discharged holey carbon film and froze in liquid ethane using a cryo-TEM sample preparation system (Vitrobot, FEI, USA). The samples were observed using a cryo-TEM (Talos F200C, FEI, USA) at −185 °C.

### 4.10 Molecular dynamics simulation

MD simulations were performed using the GROMACS software package with CHARMM36 force field and TIP3P water model. Glycosylated spike protein (PDB ID: 6VXX) was been used and the viral envelope was generated by CHARMM-GUI. The complete model includes the viral envelope, spike protein, 40S_Lip, and an aqueous layer, corresponding to about 3.87 million atoms. The Particle-Mesh-Ewald algorithm was utilized for the calculation of the electrostatic force, and the LINCS algorithm was used for the calculation of bond constraints in protein molecules. The V-rescale temperature coupling algorithm with a time constant of 0.1 ps and the C-rescale pressure coupling algorithm with a time constant of 2.0 ps was adopted in the work to maintain the system temperature at 310 K and 1.01 bar. The time step of the simulation was 2 fs. The COM distance between the 40S_Lip and S protein was set as 15.8 nm–24 nm with 84 sampling windows. Each window was first run under the NPT ensemble for 500 ps, and then under NVT ensemble sampling for 200 ps. This work was performed on a personal workstation with GTX2080super and with a computation speed of 1.2 ns/day. The WHAM tool from GROMACS was utilized for post-processing to obtain the potential of mean force (PMF) profiles.

### 4.11 Circular dichroism (CD) spectrum

One hundred microliter per milliliter 2019-nCoV spike protein RBD was incubated with 1 mg/mL 40S_Lip at 37 °C for 1 h, then the CD spectrum of spike protein was studied by a circular dichroism spectroscopy (JASCO).

### 4.12 Enzyme-linked immunosorbent assay (ELISA)

Ten million TU per milliliter wild-type SARS-CoV-2 pseudovirus was incubated with different concentration of the nanodecoys or heparin at 37 °C for 1 h. Then the amount of the RBD of spike protein was studied using a SARS-CoV-2 spike protein S1 RBD ELISA kit, the proportion of detected protein was calculated according to the formula: Detected protein (%) = [Protein Concentration (Sample) -Protein Concentration (Blank) ]/[Protein Concentration (Control) - Protein Concentration (Blank)] × 100%

### 4.13 Statistical analysis

Data was expressed as mean ± SD. The one-way ANOVA analysis was used to determine the statistical significance using no significance: n. s., *P < 0.05, **P < 0.01, ***P < 0.001.

## Ethics approval and consent to participate

This study does not perform any experiments on animals. All performed examinations and samples collection did not require local ethics committee approval.

## Declaration of interests

The authors declare that they have no known competing financial interests or personal relationships that could have appeared to influence the work reported in this paper.

## CRediT authorship contribution statement

**Xu Li:** Conceptualization, Data curation, Formal analysis, Investigation, Methodology, Project administration, Validation, Visualization, Writing – original draft, Writing – review & editing. **Ningtao Cheng:** Writing – original draft, Writing – review & editing. **Danrong Shi:** Data curation, Formal analysis, Investigation, Methodology. **Yutong Li:** Data curation, Formal analysis, Investigation. **Chen Li:** Data curation, Formal analysis, Investigation, Methodology. **Miaojin Zhu:** Investigation. **Qiao Jin:** Resources, Supervision, Validation, Writing – original draft, Writing – review & editing. **Zhigang Wu:** Investigation. **Linwei Zhu:** Investigation. **Yi He:** Resources, Supervision. **Hangping Yao:** Funding acquisition, Resources, Supervision. **Jian Ji:** Funding acquisition, Resources, Supervision.
