# Supplementary material for: Sulfated liposome-based artificial cell membrane glycocalyx nanodecoys for coronavirus inactivation by membrane fusion
Source: Bioact Mater. 2023 Nov 4;33:1–13. doi: 10.1016/j.bioactmat.2023.10.021 (PMC10660003; doi:10.1016/j.bioactmat.2023.10.021)
Supplement: Multimedia component 1 [file mmc1.docx]

**Supporting Information**

**Sulfated liposome-based artificial cell membrane nanodecoys for broad-spectrum coronavirus inactivation by membrane fusion**

Xu Li^1‡^, Ningtao Cheng^2‡^, Danrong Shi^3‡^, Yutong Li^1^, Chen Li^4^, Miaojin Zhu^3^, Qiao Jin^1^, Zhigang Wu^3^, Linwei Zhu^3^, Yi He^4^, Hangping Yao^3*^, and Jian Ji1^5*^

1 MOE Key Laboratory of Macromolecule Synthesis and Functionalization, Department of Polymer Science and Engineering, Zhejiang University, Hangzhou 310027, China

2 School of Medicine, Zhejiang University, Hangzhou 310058, China

3 State Key Laboratory for Diagnosis and Treatment of Infectious Diseases, National Clinical Research Center for Infectious Diseases, Collaborative Innovation Center for Diagnosis and Treatment of Infectious Diseases, The First Affiliated Hospital, School of Medicine, Zhejiang University, Hangzhou 310003, China

4 College of Chemical and Biological Engineering, Zhejiang University, Hangzhou 310027, China

5 Shanxi-Zheda Institute of Advanced Materials and Chemical Engineering, Taiyuan, 030032, China

***Corresponding author.**

Email: jijian@zju.edu.cn (J.J.); yaohangping@zju.edu.cn (H.Y.)

**‡ These authors contributed equally to this work**


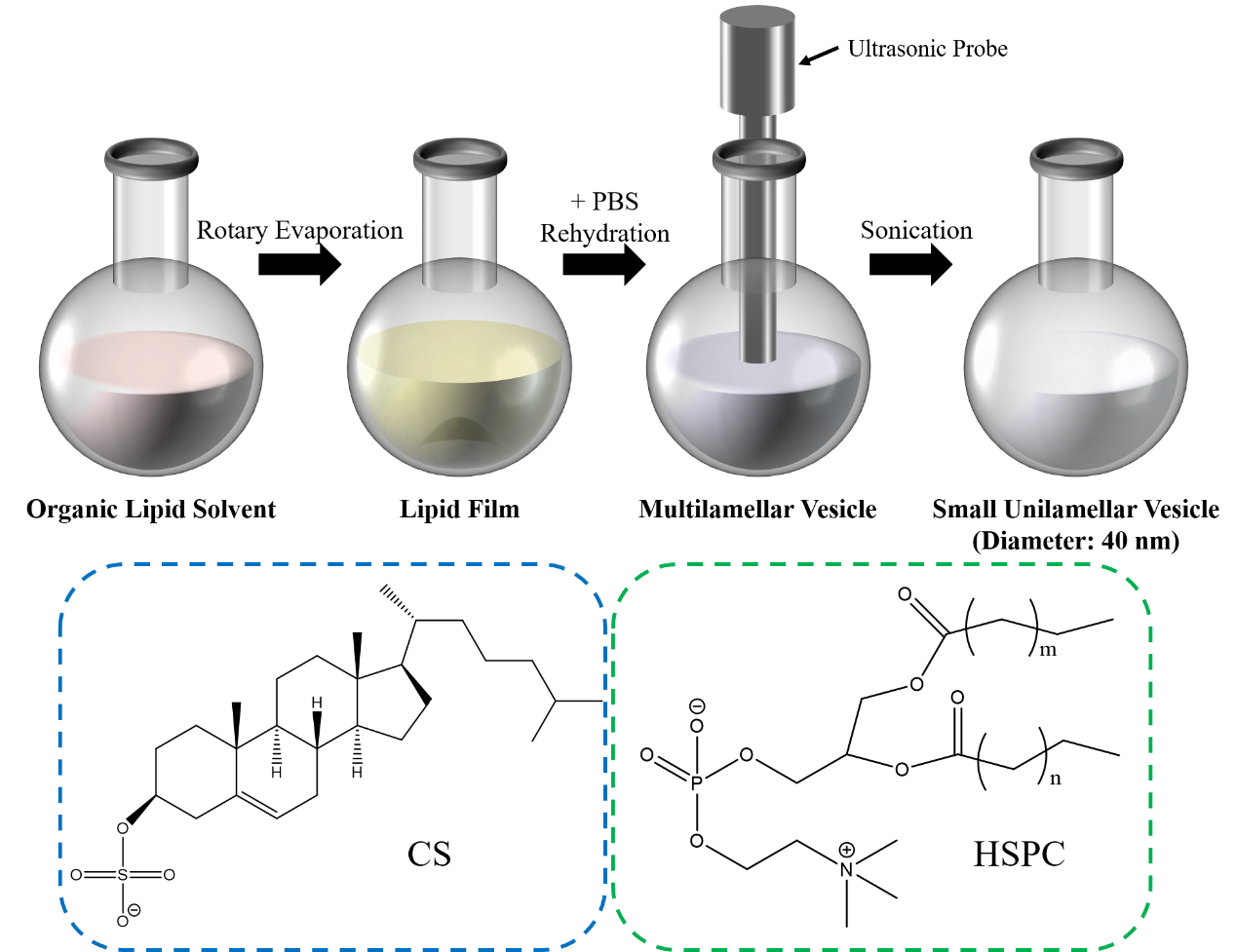


**Fig. S1.** Schematic depiction of the preparation process of the small-sized sulfonated liposome and the chemical structure of Hydrogenated soy phosphatidylcholine (HSPC) and sodium cholesteryl sulfate (CS). The small-sized liposome was prepared by the filming-rehydration method. Firstly the lipid was dissolved by the organic solvent and transferred to a round-bottom flask. Then the organic solvent was removed by rotary evaporation, and the lipid dissolved in the solvent would form a lipid film on the inner surface of the flask. The phosphate buffer saline (PBS) was added to the flask, after shaking the flask for a while, the film would be peeled off from the surface of the flask and the multilamellar vesicle would be formed. By sonicating with the ultrasonic probe, the small-sized liposome with a diameter of 40 nm would be obtained.


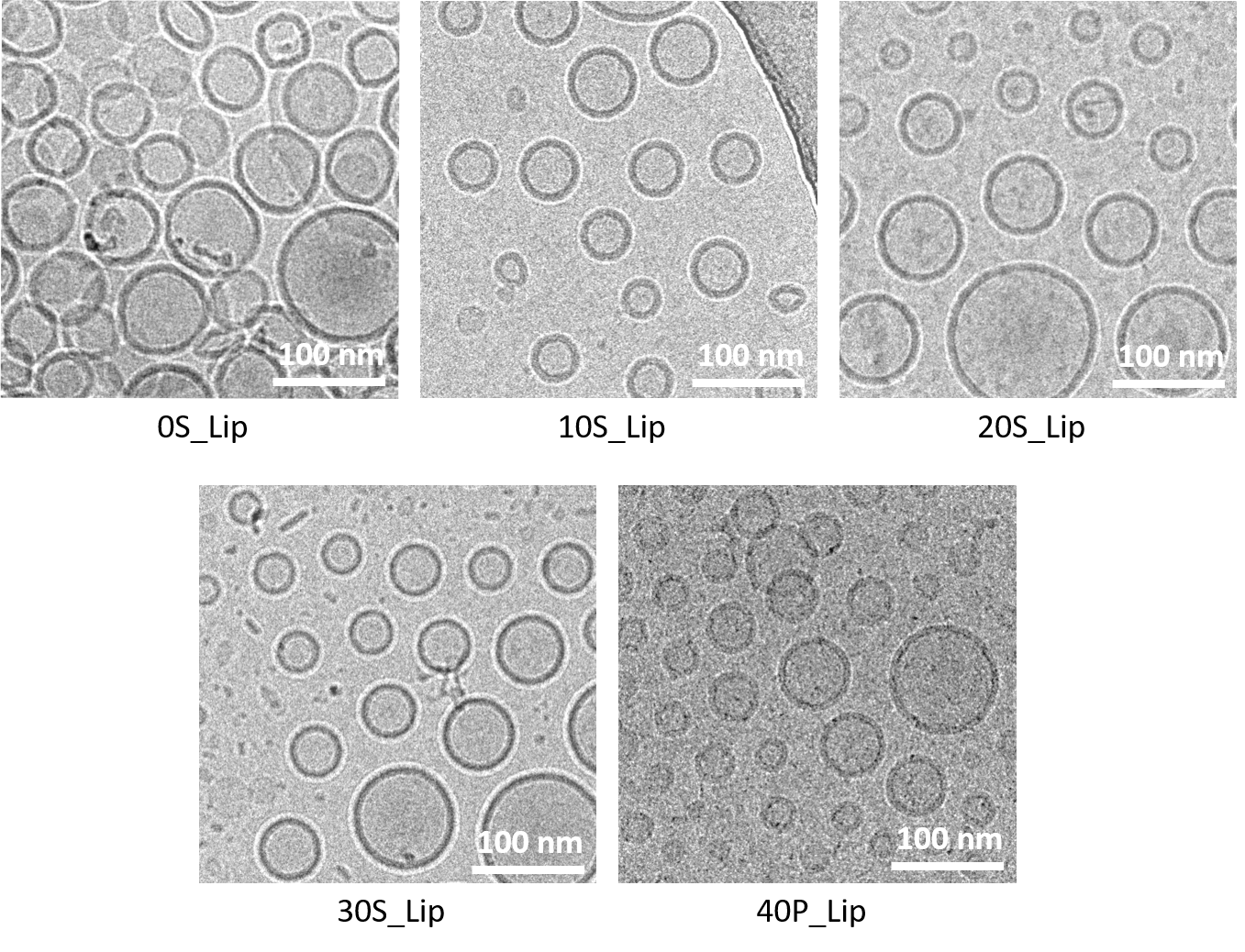


**Fig. S2.** The cryo-TEM picture of nanodecoys.


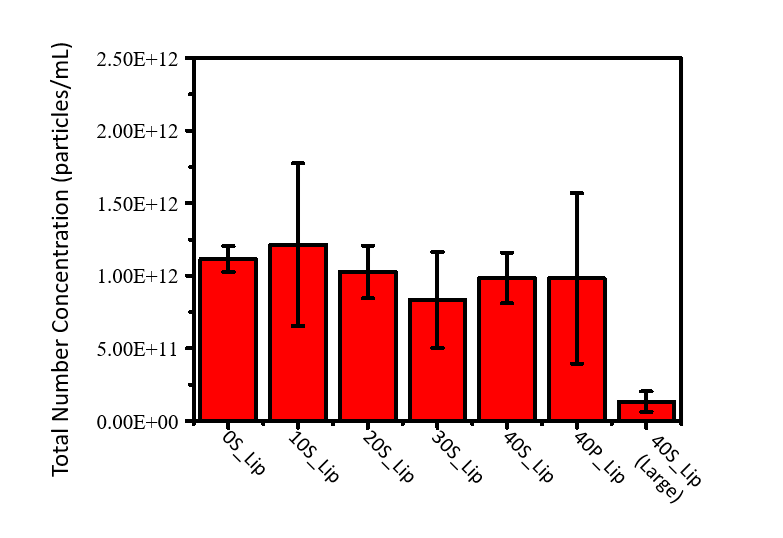


**Fig. S3.** The particle concentration of the nanodecoys.

**
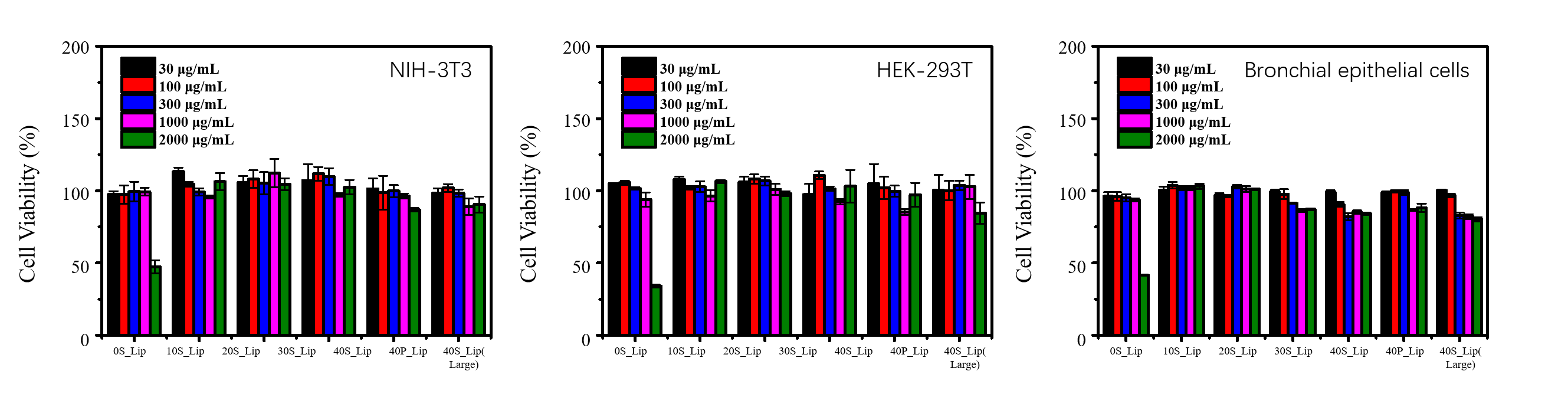
Fig. S4.** The cell viability of NIH-3T3, HEK-293T, and bronchial epithelial cells after incubating with nanodecoys with different concentrations.


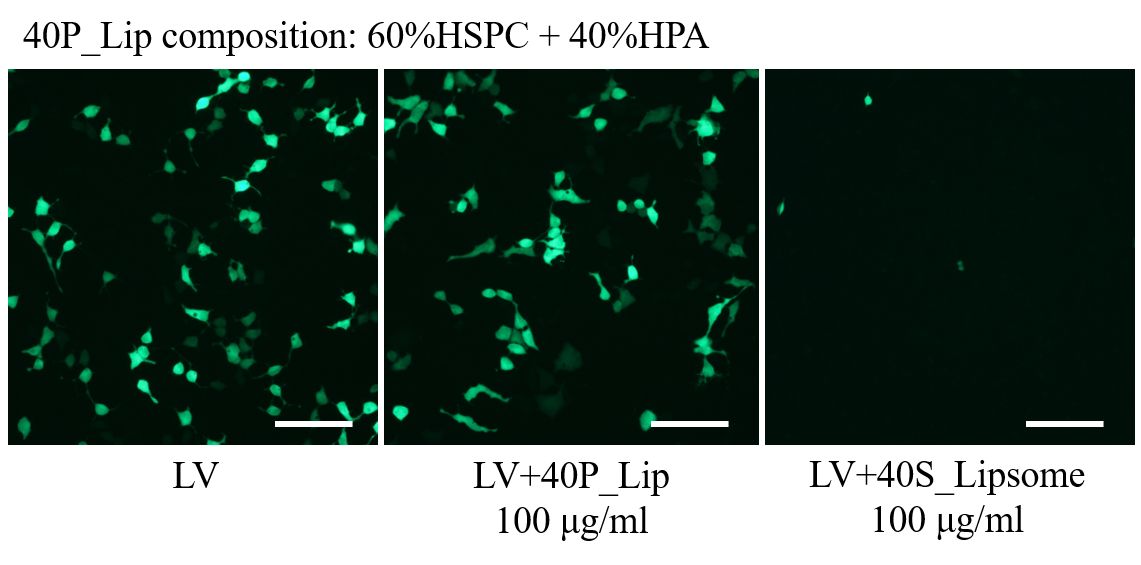


**Fig. S5.** The fluorescent picture of the HEK-293T cell after being infected by the lentivirus incubated with 100 μg/mL 40P_Lip or 40S_Lip


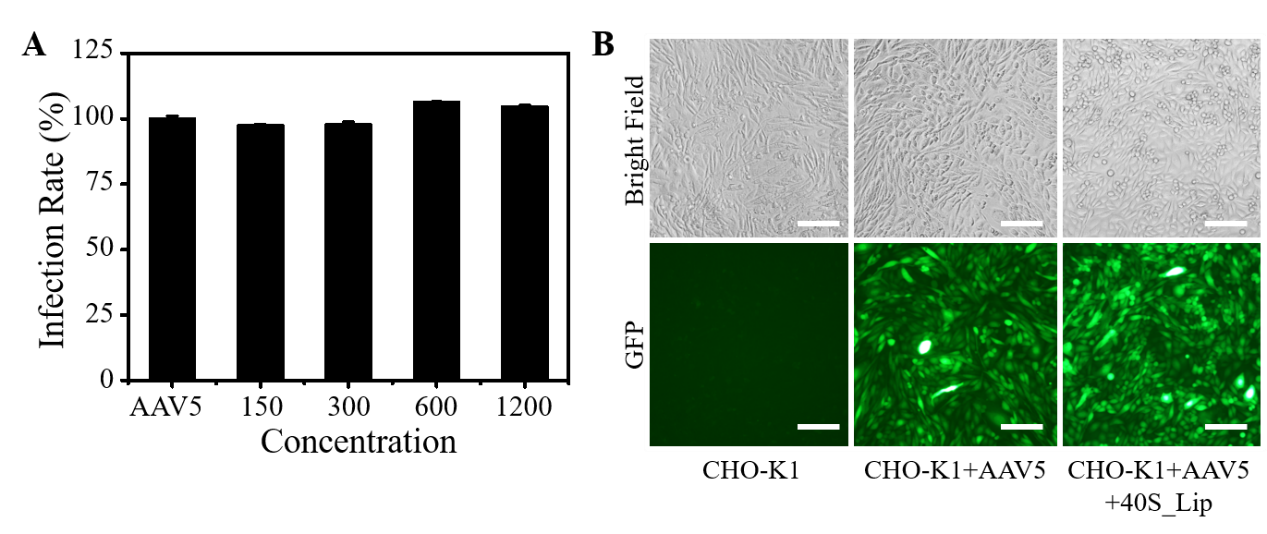


**Fig. S6.** (A) The infection rate of the AAV5 was incubated with different concentrations of 40S_Lip. (B) The fluorescent picture of the CHO-K1 cell after being infected by the AAV5 incubated with 1200 μg/mL 40S_Lip.


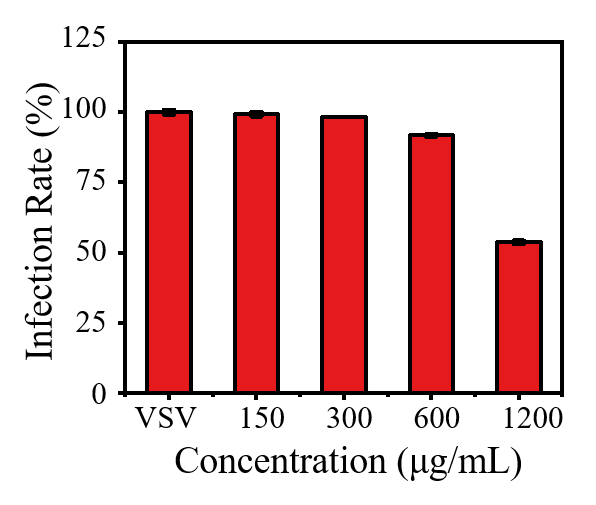


**Fig. S7.** The infection rate of VSV incubated with different concentration of 40S_Lip.


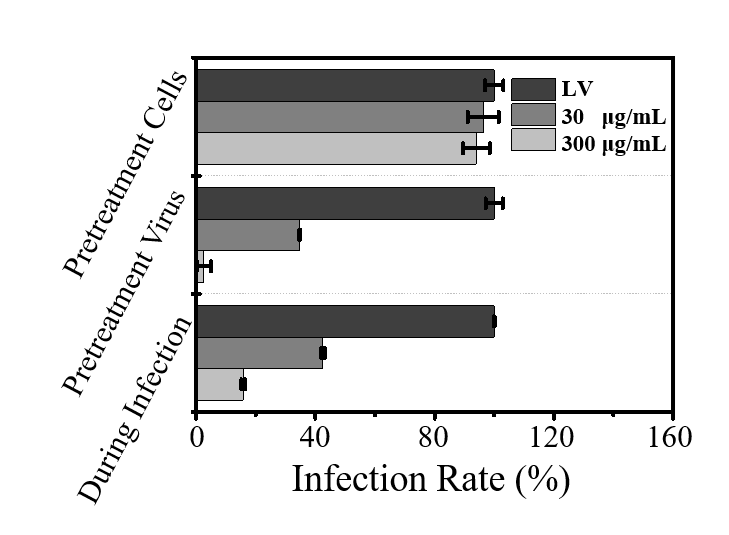


**Fig. S8.** The time-of-addition assay result of 40S_Lip. Pretreatment Cell means the cell was pre-incubated with 40S_Lip for 1 h and add the lentivirus in after removing the 40S_Lip and washing with PBS 3 times. Pretreatment Virus means that the virus was pre-incubated with 40S_Lip for 1 h before being added to cells. During Infection means that the lentivirus and the 40S_Lip were added to the cell at the same time.


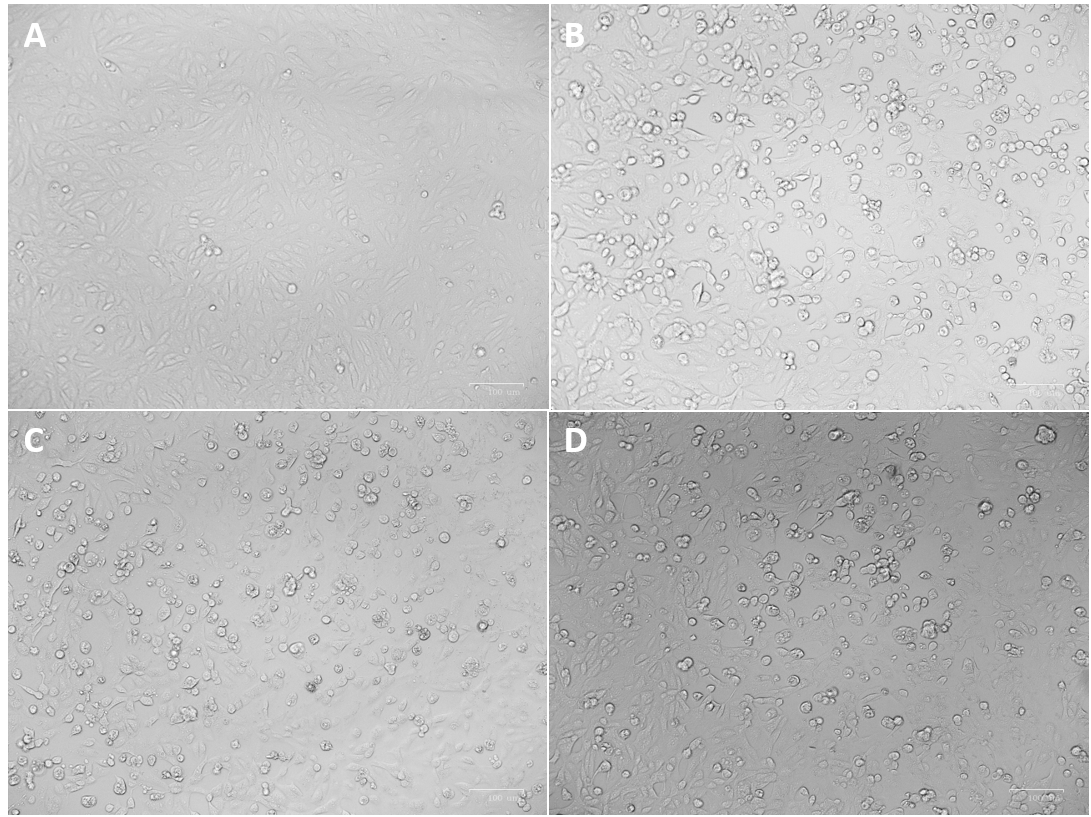


**Fig. S9.** The micrograph of Vero cells after incubation with (A) PBS, (B) SARS-CoV-2 or (C) SARS-CoV-2 and 900 μg mL-1 0S_Lip or (D) SARS-CoV-2 and 900 μg ml-1 40P_Lip to reveal the cytopathic effect (CPE). Scale bar: 100 μm.


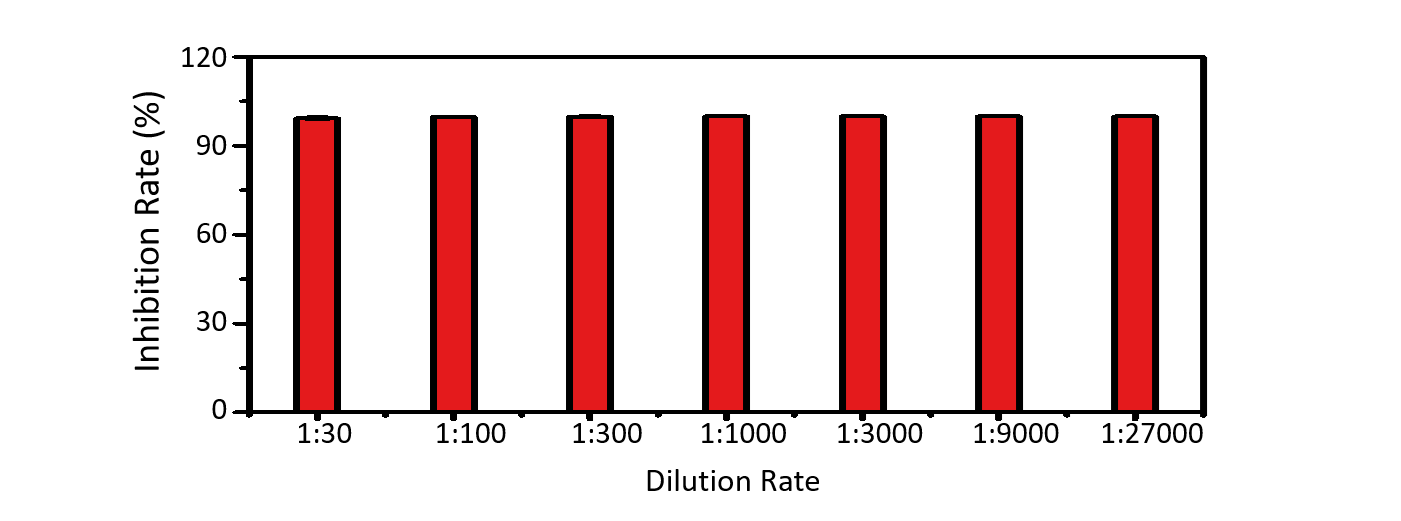


**Fig.S10.** The inhibition rate of 900 μg ml-1 40S_Lip incubated with 10E6 TCID50/ml SARS-CoV-2 of different dilution rate.


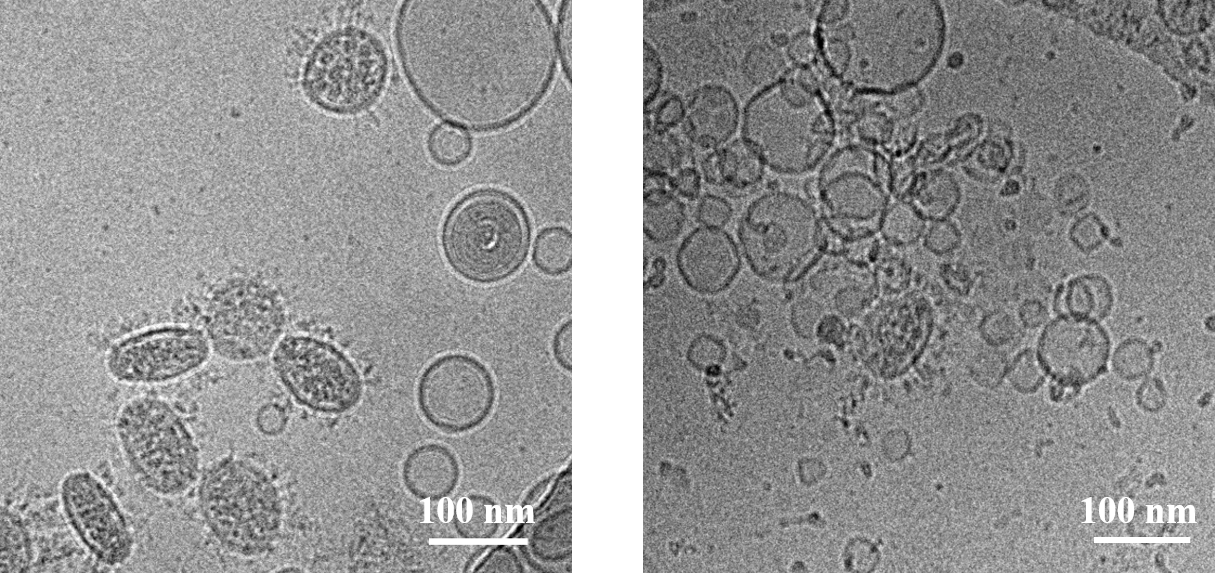


**Fig. S11.** The cryo-TEM image of the virus incubated with 40S_Lip(Large) (left) and 0S_Lip (right). Scale bar: 100 nm.


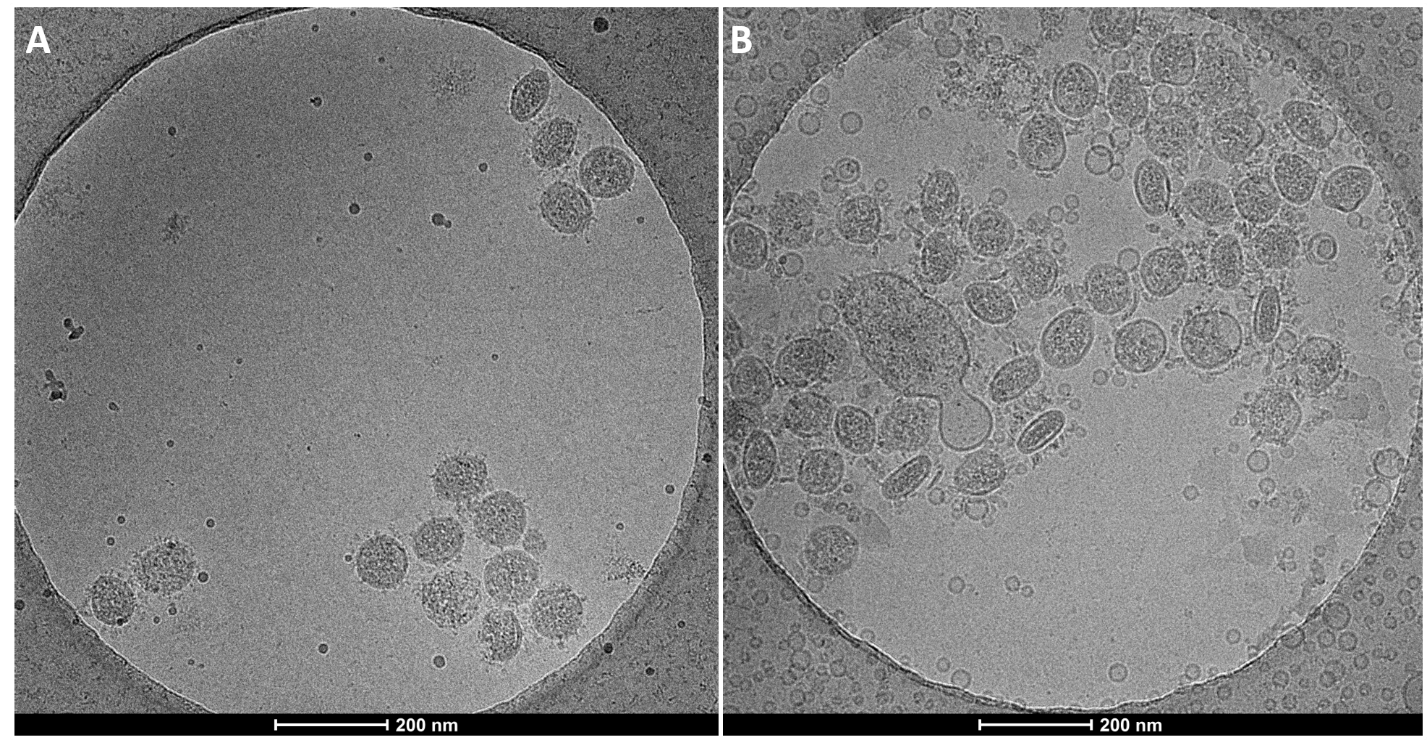


**Fig. S12.** The large scalge cryo-TEM picture of (A) SARS-CoV-2 and (B) SARS-CoV-2 incubated with 40S_Lip.


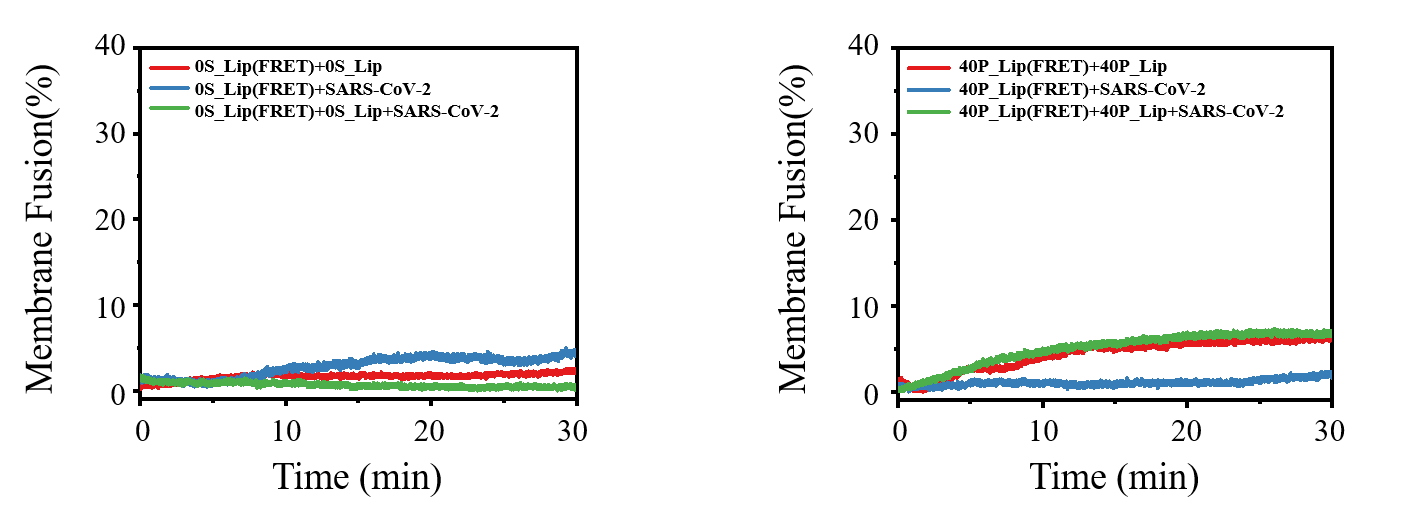


**Fig. S13.** The membrane fusion profile of the FRET lipid labeled 0S_Lip(FRET) and 40P_Lip(FRET) incubated with no FRET lipid labeled nanodecoy, SARS-CoV-2 pseudovirus, or 40S_Lip and SARS-CoV-2 pseudovirus.

**Fig. S14.** Histograms of the umbrella sampling. The PMF curves were calculated from this data. The unsampled part of the PMF is obtained by interpolation of the curve obtained from WHAM.
